# Comment on: The experience of pregnancy in the COVID-19 pandemic

**DOI:** 10.61622/rbgo/2025rbgo49

**Published:** 2025-07-15

**Authors:** Bibek Roy

**Affiliations:** 1 Lovely Professional University Phagwāra Punjab India Lovely Professional University, Phagwāra, Punjab, India.

I read with interest the article by Lopes et al. entitled "The Experience of Pregnancy in the COVID-19 Pandemic".^(1)^ The topic is timely and important, and the authors deserve appreciation for exploring various maternal experiences in such a complex context. However, I would like to respectfully draw attention to some methodological and scientific issues that could compromise the validity and interpretation of the work.

First, the sample was limited to women who were receiving care in private facilities, and most of them had higher incomes and levels of education. In a nation with significant health disparities, this creates socioeconomic homogeneity, which significantly reduces the study's representativeness. Furthermore, the scope is further limited and selection bias is done by excluding postpartum women who tested positive for COVID-19, who are arguably one of the most affected categories.

Second, even though the authors say they employ Krippendorff's Content Analysis,^(2)^ this approach is more commonly linked to quantitative coding schemes and isn't the best fit for the study's subjective investigation. Additionally, there is a gap between the suggested and implemented approaches since the method's analytical features like dendrograms are missing.

Third, without using methodological safeguards like data triangulation or saturation tracking, which are crucial for qualitative rigor, the study makes a number of claims regarding the pandemic's effect on pregnancy experiences. The reliance on virtual interaction as a common factor was discussed with insufficient attention to Brazil's digital divide, which may have left out important viewpoints.

Lastly, although the research finds that its findings are consistent with those of other studies, it fails to consider Brazil's distinct maternal health system structure, which may have a significant impact on experiences across various socioeconomic strata. I believe these issues warrant clarification to avoid misinterpretation and to uphold the scientific rigor expected of publications in RBGO. With regard to study design and representation in prenatal mental health studies, I believe my letter makes a positive contribution to the current conversations.^(1)^
