# Supplementary material for: Comment on: The experience of pregnancy in the COVID-19 pandemic
Source: Rev Bras Ginecol Obstet. 2025 Jul 15;47:e-rbgo49. doi: 10.61622/rbgo/2025rbgo49 (PMC12266852; doi:10.61622/rbgo/2025rbgo49)
Supplement: Related texts

# Author's reply to comment on: The Experience of Pregnancy in the COVID-19 Pandemic

**Authors:** Mariana Corniani Lopes, Cheryl Tatano Beck, Zelina Hilária de Souza Rosa, Erika de Sá Vieira Abuchaim

**Affiliations:** 1 Universidade Federal de São Paulo Escola Paulista de Enfermagem São Paulo SP Brazil Escola Paulista de Enfermagem, Universidade Federal de São Paulo, São Paulo, SP, Brazil.; 2 University of Connecticut School of Nursing Connecticut United States University of Connecticut, School of Nursing, Storrs, Connecticut, United States.

We appreciate the reader's feedback on our article, which was based on research guided by professors from the Federal University of São Paulo and the University of Connecticut. The study aims to reveal women's experiences of pregnancy during the COVID-19 pandemic.

The authors acknowledge the socio-economic and cultural disparities faced by pregnant and postpartum women in Brazil. The vast majority of studies on the experience of Brazilian women regarding care and/or living in the pregnancy-puerperal cycle are carried out in public health services, a situation that can also be considered a limitation. The opportunity to understand the experiences of women receiving assistance in private health institutions is particularly significant, especially during the pandemic when concerns about insufficient knowledge, human resources, materials, and technical support were major stress factors. The pandemic has impacted women from all social strata. During this period, the significance attributed to this experience is unique, as the institution restricted contact with infected women due to isolation measures and local restrictions. Therefore, we conducted interviews with postpartum women hospitalized in the postpartum room.

As the objectives are clarified for the reader regarding the nature of the study, two theoretical and methodological references are provided. The demonstration (including limitation, vision, and representation), the presentation of the results, and the discussion of two common aspects for those unfamiliar with qualitative research, aimed at comprehending its delineation compared to quantitative methods. This is pertinent especially when the adopted methodological reference, such as Krippendorff's Content Analysis,^(1,2)^ can be applied to both qualitative and quantitative approaches. Even within the qualitative approach, this can be achieved through categorical or thematic analysis, with the latter being the model utilized in this study.

While this method is less commonly used in Brazil, it is a well-established and rigorously validated approach in Europe and the U.S., ensuring both truth-worthiness and reliability in our findings. We applied systematic thematic analysis in strict adherence to Krippendorff's framework, a process that prioritizes methodological transparency and minimizes bias. The resulting thematic patterns, shown in the dendrograms (pages 4–6), were created from a structured, iterative analytical process, further reinforcing the study's truth-worthiness by demonstrating clear, reproducible patterns in the data. It is important to emphasize that a representative sample, with the aim of achieving generalizable results, is a fundamental characteristic of quantitative research.

Qualitative studies that adopt Krippendorff's Content Analysis seek to understand the meanings of the symbols of a given phenomenon within the context in which it was produced and are not subject to generalization. The aim was to understand the experiences of postpartum women hospitalized in the rooming-in unit during pregnancy, examining them from their perspective and considering the context of the pandemic and not the disease itself or its impacts.

Not that it involves demonstration, studies of a qualitative nature adopt non-probabilistic demonstration outlines, such as, for example, demonstration by convenience used in the present study, being endorsed by means of its adequacy and appropriation.

The final sample is generally small and usually defined over the length of the study to guarantee the satisfaction and depth of the information perceived. Theoretical saturation, provided by this study, occurs when even new data does not reveal new information and the redundancy was reached. As presented in our article, even though the saturation of the data has been determined before the analysis of the 106 interviews, we opted for the analysis of all material, ensuring methodological rigor.

The thematic representation, described in a hierarchical manner, demonstrating the construction of reasoning by researchers and adhering to Krippendorff's proposed methodology, was presented in manuscript by means of four tree diagrams called dendrograms on pages 4-6.

We are available to assist with any questions regarding the drafting of a qualitative study and are ready to clarify any remaining doubts.

## References

[1] Krippendorff K (2019). Content analysis: an introduction to its methodology.

[2] Rossi GB, Serralvo FA, Joao BN (2014). Análise de conteúdo. ReMark Rev Bras Mark.
